# High Levels of Heterogeneity in the HIV Cascade of Care across Different Population Subgroups in British Columbia, Canada

**DOI:** 10.1371/journal.pone.0115277

**Published:** 2014-12-26

**Authors:** Lillian Lourenço, Guillaume Colley, Bohdan Nosyk, Dmitry Shopin, Julio S. G. Montaner, Viviane D. Lima, on behalf of the STOP HIV/AIDS Study Group

**Affiliations:** 1 British Columbia Centre for Excellence in HIV/AIDS, Vancouver, Canada; 2 Faculty of Health Sciences, Simon Fraser University, Burnaby, Canada; 3 Faculty of Medicine, University of British Columbia, Vancouver, Canada; University of Athens, Medical School, Greece

## Abstract

**Background:**

The HIV cascade of care (cascade) is a comprehensive tool which identifies attrition along the HIV care continuum. We executed analyses to explicate heterogeneity in the cascade across key strata, as well as identify predictors of attrition across stages of the cascade.

**Methods:**

Using linked individual-level data for the population of HIV-positive individuals in BC, we considered the 2011 calendar year, including individuals diagnosed at least 6 months prior, and excluding individuals that died or were lost to follow-up before January 1^st^, 2011. We defined five stages in the cascade framework: HIV ‘diagnosed’, ‘linked’ to care, ‘retained’ in care, ‘on HAART’ and virologically ‘suppressed’. We stratified the cascade by sex, age, risk category, and regional health authority. Finally, multiple logistic regression models were built to predict attrition across each stage of the cascade, adjusting for stratification variables.

**Results:**

We identified 7621 HIV diagnosed individuals during the study period; 80% were male and 5% were <30, 17% 30–39, 37% 40–49 and 40% were ≥50 years. Of these, 32% were MSM, 28% IDU, 8% MSM/IDU, 12% heterosexual, and 20% other. Overall, 85% of individuals ‘on HAART’ were ‘suppressed’; however, this proportion ranged from 60%–93% in our various stratifications. Most individuals, in all subgroups, were lost between the stages: ‘linked’ to ‘retained’ and ‘on HAART’ to ‘suppressed’. Subgroups with the highest attrition between these stages included females and individuals <30 years (regardless of transmission risk group). IDUs experienced the greatest attrition of all subgroups. Logistic regression results found extensive statistically significant heterogeneity in attrition across the cascade between subgroups and regional health authorities.

**Conclusions:**

We found that extensive heterogeneity in attrition existed across subgroups and regional health authorities along the HIV cascade of care in B.C., Canada. Our results provide critical information to optimize engagement in care and health service delivery.

## Introduction

Since the introduction of modern highly active antiretroviral therapy (HAART), people living with HIV are living longer and healthier lives than ever before [1], [2]. Immediate use of HAART has been shown to delay progression to AIDS and subsequent HIV-related mortality [3], [4], [5]. A 20-year-old individual diagnosed with HIV infection today, would be expected to live an additional five decades, on life-long HAART [6]. In recent years, HAART has been shown to have a secondary population-level benefit of reducing the risk of onward HIV transmission. The concept of “Treatment as Prevention (TasP)” has been supported by ecologic, randomized control trials, mathematical modelling, and cohort studies [7], [8], [9], [10], [11], [12]. Definitive evidence of the magnitude of the impact of HAART on HIV transmission comes from HPTN052 [7]. This randomized control trial found that immediate, rather than delayed initiation of HAART in HIV-positive individuals within serodiscordant heterosexual couples reduced the risk of HIV transmission by 96%. More recently, the preliminary results of the PARTNER study provided evidence suggesting that this effect is similarly present among homosexual couples [13].

For the benefits of HAART to be fully realized (both at the individual and population-level), HIV infected individuals must be fully engaged in the ‘HIV continuum of care’. That is, they must be aware of their HIV infection, linked and retained in HIV care, initiate HAART and achieve high levels of adherence to achieve and sustain full virologic suppression. Incomplete engagement at any of the stages in the HIV continuum of care will compromise the impact of HAART at the individual and societal levels, as determined by increased risk of HIV-related morbidity, mortality and transmission [14], [15], [16], [17], [18], [19], [20].

To this end, the HIV cascade of care (referred to as the cascade hereafter) has been proposed as a comprehensive monitoring tool to identify attrition, or ‘leakage’ points, along the ‘HIV continuum of care’, and to guide programmatic efforts to improve individual health outcomes and decrease HIV transmission [21], [22]. The cascade has become a focal point in the monitoring and evaluation of TasP initiatives worldwide [23], [24], [25], [26], [27], [28], [29].

HIV does not affect a population homogeneously. Rather, subgroups share specific behaviours and they experience a variety of societal barriers, which may impact how HIV care is both accessed and accepted. It has therefore been suggested that the cascade can become increasingly informative when heterogeneity is taken into consideration [25]. Here, we apply multiple stratifications to the population-based cascade in British Columbia, Canada, by sex, age, risk group, and regional health authority to explore its heterogeneity in British Columbia (BC), Canada.

## Methods

### Data Sources

We used multiple data sources to develop a database to characterize our population-based cascade. Data linkage and preparation of the de-identified individual-level database was facilitated by the B.C. Ministry of Health and methods have been described in detail elsewhere [30], [31]. In summary, we used a series of linked provincial datasets (which includes all BC residents who were diagnosed as HIV-positive between January 1^st^, 1996 and March 31^st^, 2012) to estimate the number of known HIV-positive individuals in each stage of the cascade, as defined in Table 1. The definitions used for our cascade have been previously validated [23]. From this previously validated work, we modified the definition of viral load suppression from a detection threshold of <50 copies/mL to <200 copies/mL, which removes the effect of viral load assay variability. The B.C. Centre for Disease Control (BCCDC) provided HIV testing and diagnosis data. The BCCDC is the single provincial agency which centralises all HIV testing and new diagnosis reports from the B.C. Public Health Microbiology and Reference Laboratory, which performs all confirmatory HIV testing in the province. Reporting of new HIV diagnoses is mandatory in B.C. since 2003; however, reporting can be done either nominally or non-nominally.

**Table 1:** HIV Cascade of Care Definitions.

Cascade Stage	Timeframe	Definition
HIV ‘diagnosed’	Study participants were diagnosed with HIV for ≥18 months before January 1^st^, 2012.	The first instance of one of the following: a confirmed HIV-positive test, a detectable viral load ≥50 copies/mL, an HIV-related physician visit or hospitalization, a reported AIDS-defining illness or the dispensation of antiretroviral treatment.
‘Linked’ to HIV care	Linkage can happen any time before or during 2011.	i) Among HIV diagnosed individuals (*with* a confirmed HIV test): the first instance of an HIV-related service (i.e., a plasma viral load test, CD4 cell count, HIV-related physician visit, or antiretroviral treatment dispensed) following HIV diagnosis; or ii) Among HIV diagnosed individuals (*without* a confirmed HIV test): the first instance of an HIV-related service ≥30 days following the derived HIV diagnosis date.
‘Retained’ in HIV care	Retained in the year 2011.	Among persons linked to HIV care, individuals who had: i)An HIV-related physician visit or a diagnostic test (CD4 cell count or plasma viral load test) ≥3 months apart in 2011; or ii) At least two HAART dispensations ≥3 months apart within 2011.
‘On HAART’	On HAART in the year 2011.	Among individuals retained in HIV care, individuals who had at least two HAART drug dispensations ≥3 months apart in 2011.
Virologically ‘suppressed’	Suppressed in the year 2011.	Among those on HAART, individuals with at least one episode ≥3 months long of an undetectable plasma viral load (i.e., <200 copies/mL) in 2011.

The BC Centre for Excellence in HIV/AIDS (BC-CfE) provided plasma viral load, CD4 cell count, and HAART prescription data, which were used to calculate the cascade stages linked to HIV care, retained in HIV care, on HAART, and virologically suppressed. The BC-CfE is the central agency responsible for provincial distribution of HAART [32]. Of note, the BC-CfE captures 100% of plasma viral load data, as well as approximately 85% of CD4 cell count testing for the province. Related medical and laboratory monitoring and HAART are free of charge (no co-payments or deductibles) to all HIV-infected B.C. residents.

The above data were supplemented with linkages to the B.C. Ministry of Health databases, including: the Medical Services Plan (MSP) physician billing data; the Discharge Abstract Database, which records inpatient care; PharmaNet, which captures non-antiretroviral drug dispensations; and the B.C. Vital Statistics Database, which provides mortality data.

### Eligible Individuals

We included all individuals (regardless of age) in our linked dataset who were diagnosed with HIV at least 18 months before January 1^st^, 2012. We excluded all those who died or met our criteria for administrative loss to follow-up (defined as having no record of death or health administrative records from any of the linked databases for a period of at least 18 months) before January 1^st^, 2011. We followed individuals until the end of 2011.

### Stratification of the HIV Cascade of Care

We stratified the cascade by various socio-demographic, including sex (male, female); age as of January 1^st^, 2011 (categorized as <30, 30–39, 40–49 and ≥50 years); and risk group categorized as men who have sex with men (MSM), having a history of injection drug use (IDU), both MSM and IDU (MSM/IDU), heterosexual, or other (blood transfusion or perinatal exposure or unknown risk group (i.e., risk factor not requested or identified)). For the MSM and IDU groups, we also stratified their cascade by age and gender (where applicable). Finally, we explored geographic variation by developing stratified cascades for each of B.C.'s five regional health authorities (HAs). BC's five HAs-Vancouver Coastal HA (VCHA), Interior HA, Vancouver Island HA (VIHA), Northern HA, and Fraser HA-are responsible for the management and care of HIV-positive individuals in their region. In these cascades, we reported leakage by calculating the ‘percent loss’ between each of its steps.

### Multiple regression analysis of factors associated with attrition along the cascade

Multiple logistic regression models were created to predict attrition across stages of the cascade. Models were adjusted for age, sex, risk, and HA. Variables associated with each outcome at the bivariate level (p<0.05) were entered into the multivariate adjusted logistic regression models. Odds ratios are reported at 95% confidence intervals. The analysis was completed using SAS software version 9.3 (SAS, Cary, NC).

### Ethics Statement

The BC-CFE received approval for this study from the University of British Columbia ethics review committee at the St Paul's Hospital, Providence Health Care site (P05–123). The study complies with the BC's Freedom of Information and Protection of Privacy Act. The study was conducted primarily using anonymized laboratory and administrative databases, and therefore informed consent was not required. Incidence data were augmented with data collected through prospective research cohort studies, which include written informed consent by study participants and separate IRB approval.

## Results

**Figure 1:**
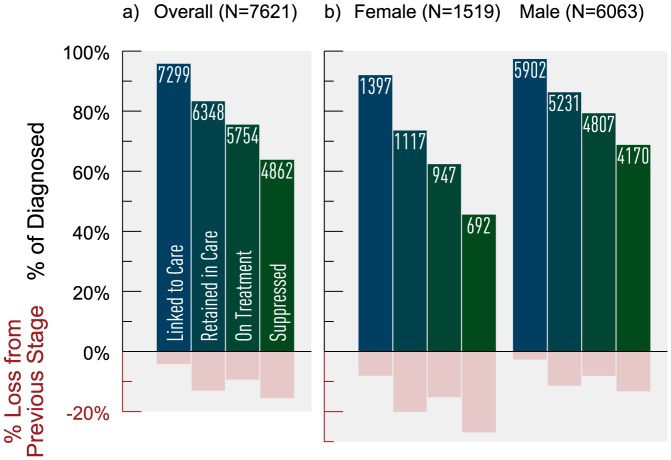
Estimated HIV cascade of care overall and by sex in 2011 for British Columbia. *There were 39 HIV-diagnosed individuals with missing sex information; none of these individuals were linked to HIV care.

A total of 7621 individuals were eligible for our analyses. Of the total study population, 80% (N = 6063) were male; 5% (N = 405) were aged <30 years, 17% (N = 1275) 30–39 years, 37% (N = 2843) 40–49 years, 40% (N = 3059) ≥50 years and in 1% (N = 39) age was missing. Additionally, 32% (N = 2465) of individuals self-identified as MSM, 28% (N = 2095) as IDU, 8% (N = 629) as MSM/IDU, 12% (N = 930) as heterosexual, and 20% (N = 1502) as other. Of the 7621 diagnosed individuals, 96% (N = 7299) were linked to care, 87% of linked individuals (N = 6348) were retained in care, 91% (N = 5754) of retained individuals were on HAART and 85% (N = 4862) of individuals on HAART were suppressed (Fig. 1).

Stratifying the cascade by sex revealed that females experienced the greatest attrition along the cascade (Fig. 1). A smaller proportion of females on HAART achieved virologic suppression compared with males (73% vs. 87%). Greatest areas of attrition for both females and males were in the transitions from linked to retained (20% loss for females vs. 11% loss for males), retained to on HAART (15% loss for females vs. 8% loss for males) and adherent to suppressed (27% loss for females vs.13% loss for males).

**Figure 2:**
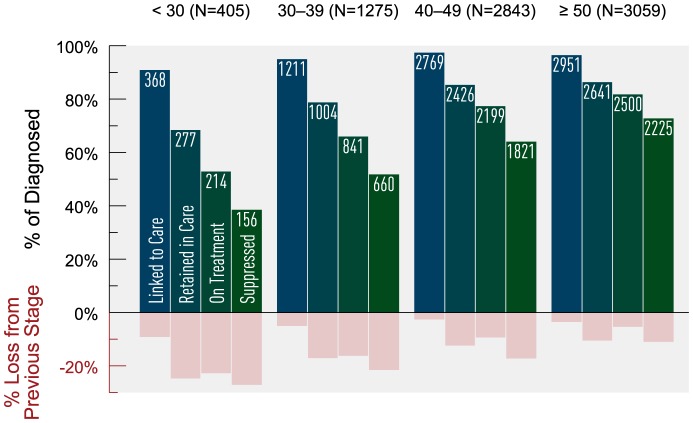
Estimated HIV cascade of care by age category in 2011 for British Columbia. * Age is defined as age as of January 1^st^, 2011. There were 39 HIV-diagnosed individuals with missing age information; none of these individuals were linked to HIV care.

**Figure 3:**
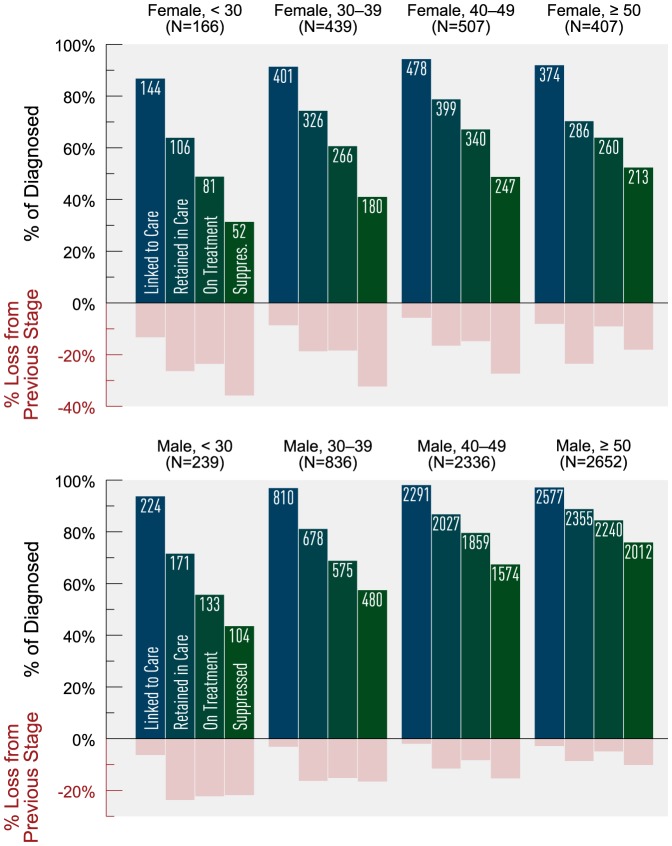
Estimated HIV cascade of care by age category and sex in 2011 for British Columbia. *Age is defined as age as of January 1^st^, 2011. There were 39 HIV-diagnosed individuals with missing gender and age information; none of these individuals were linked to HIV care.

Age stratification indicated that attrition across the cascade decreased with increasing age (Fig. 2). The youngest age group (<30 years) consistently demonstrated the greatest attrition across the cascade, with nearly 25% loss from linked to retained and only 73% of those on HAART achieving virologic suppression. Individuals ≥50 years demonstrated the least attrition overall, and they had the highest proportion of virologic suppression (89%) among those on HAART. Further stratification by age and sex showed that females experienced greater attrition than males, regardless of age category (Fig. 3).

**Figure 4:**
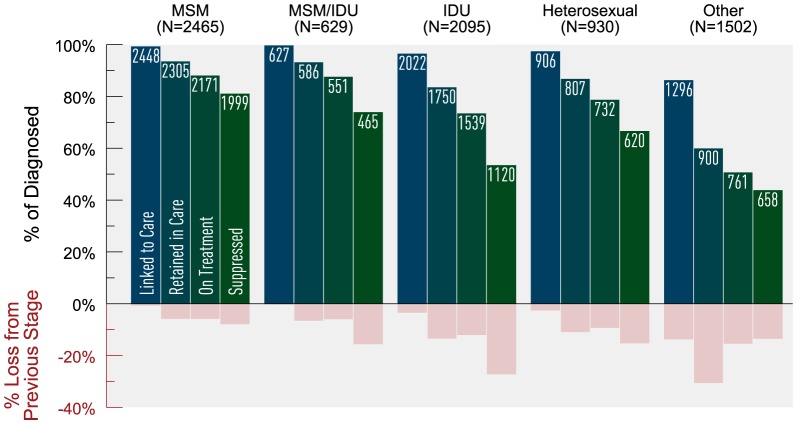
Estimated HIV cascade of care by risk category in 2011 for British Columbia. *MSM  =  men who have sex men, IDU  =  history of injection drug use, MSM/IDU  =  both MSM and IDU, Other  =  blood transfusion or perinatal exposure or unknown risk group (i.e., risk factor not requested or identified).

Stratification by transmission risk category revealed that MSM and MSM/IDUs on HAART achieved 92% and 84% virologic suppression, respectively (Fig. 4). Both groups experienced greatest attrition between on HAART and suppressed, 8% loss for MSM and 16% loss for MSM/IDUs. Overall, MSM experienced the least attrition of all risk groups. Heterosexuals and IDUs on HAART achieved 85% and 73% virologic suppression respectively and experienced the greatest attrition between the stages linked to retained (11% loss and 14% loss) and between on HAART and suppressed (15% loss and 27% loss), respectively. Those in the “other” category achieved 86% suppression (of those on HAART) and these individuals experienced the greatest attrition between linked and retained (31% loss).

**Figure 5:**
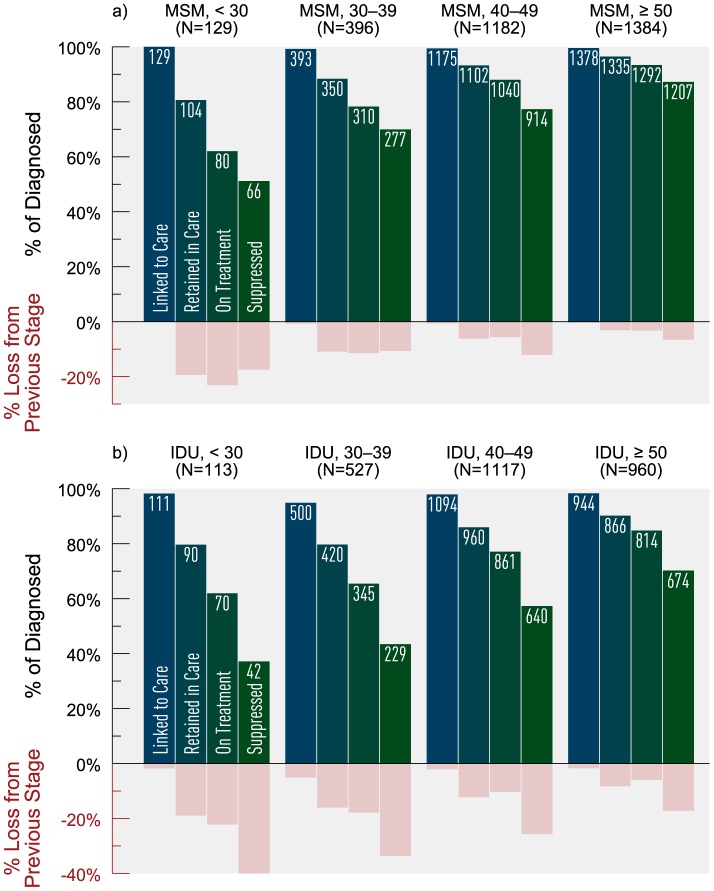
Estimated HIV cascade of care for MSM by age category and IDU by age category in 2011 for British Columbia. *MSM  =  men who have sex men; IDU  =  history of injection drug use; Age is defined as age as of January 1^st^, 2011.

**Figure 6:**
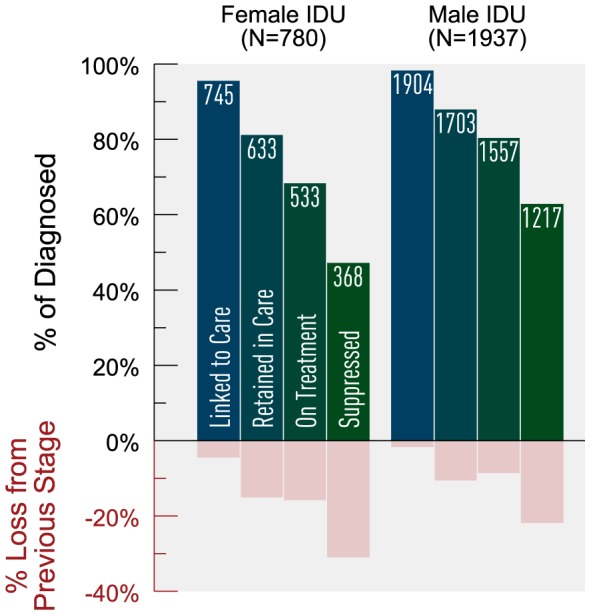
Estimated HIV cascade of care for IDU by sex in 2011 for British Columbia. *IDU  =  history of injection drug use.

We stratified the risk groups MSM and IDU by age and gender (where applicable) to further explore variances in cascade attrition. We observed increasing cascade attrition with decreasing age (Fig. 5). Young MSM on HAART had the lowest observed proportion achieving virologic suppression (83%) and experienced the greatest attrition between linked to retained (19%) and retained to on HAART (23% loss). MSM ≥50 years consistently experienced the least attrition across the cascade, and achieved the highest virologic suppression at 93% (among those on HAART). Among IDUs on HAART, virologic suppression was the lowest among those <30 years (60%) and highest among those ≥50 years (83%). Across all age groups, IDUs experienced the greatest attrition between on HAART and suppressed, with IDUs <30 years of age experiencing 40% loss between these stages. Stratifying IDU by sex revealed that females experienced greater cascade attrition compared to males, with 69% of females on HAART achieving virologic suppression versus 78% among males (Fig. 6). The greatest attrition occurred for both groups between the stages on HAART and suppressed (31% loss for females vs. 22% loss for males).

**Figure 7:**
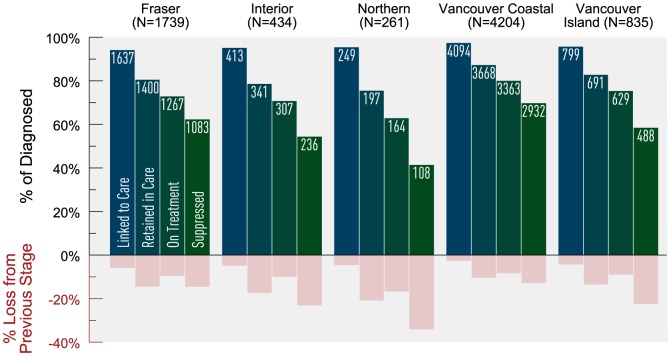
Estimated HIV cascade of care by Regional Health Authority in 2011 for British Columbia. *There were 148 HIV-diagnosed individuals with missing Regional Health Authority information. Of these, 106 were linked to care; 51 of those linked to HIV care were retained in care; 24 of those retained in care were on antiretrovirals (ARVs) and 14 of those on ARV were suppressed.

There were 148 diagnosed individuals with an unknown HA who were not included in the cascade stratification by regional HA. The Northern HA experienced the greatest level of attrition along the cascade and the least attrition occurred in the Vancouver Coastal HA (Fig. 7). Vancouver Coastal, Fraser, Interior, Vancouver Island, and Northern HAs achieved 87%, 85%, 77%, 76% and 66% virologic suppression among those on HAART, respectively. All HAs experienced the greatest attrition between the stages linked to retained and on HAART to suppressed.

### Factors associated with attrition across stages in the cascade

At the bivariate level, all study variables were significantly associated with attrition along the cascade and included in our multivariate logistic regression models (Table 2). Multiple logistic regression analysis found that, after controlling for all other factors, the odds of not being linked among diagnosed individuals were significantly higher for individuals <30 years old (compared with individuals 30–39 years old), those in the “other” risk category (compared with MSM) and residents of Northern HA (compared with residents of Vancouver Coastal HA) but significantly lower for those 40–49 and ≥50 years old (compared with those 30–39 years old) and MSM/IDUs (compared with MSM). The odds of not being retained among those linked were significantly higher for females (compared with males), individuals <30 years old (compared with individuals 30–39 years old), IDUs, heterosexuals, and those in the other risk category (compared with MSM), and residents of the Fraser and Northern HAs (compared with residents of Vancouver Coastal HA). The odds of being retained were significantly higher for those 40–49 and ≥50 years old (compared with those 30–39 years old) and for MSM/IDU (compared with MSM), controlling for all other factors.

**Table 2:** Adjusted odds ratios predicting attrition between stages of the 2011 HIV cascade of care based on sex, age, transmission risk category and regional health authority for British Columbia residents diagnosed with HIV before 2011.

	Linked		Retained		On HAART		Suppressed	
	aOR	95% CI	aOR	95% CI	aOR	95% CI	aOR	95% CI
**Sex**								
***Female***	1.00	–	1.00	–	1.00	–	1.00	–
***Male***	0.98	(0.81, 1.19)	0.79	(0.69, 0.92)	0.75	(0.65, 0.86)	0.70	(0.62, 0.80)
**Age (years)**								
***30–39***	1.00	–	1.00	–	1.00	–	1.00	–
***<30***	1.80	(1.40, 2.30)	1.86	(1.49, 2.32)	1.94	(1.56, 2.41)	2.05	(1.64, 2.58)
***40–49***	0.51	(0.42, 0.62)	0.60	(0.52, 0.71)	0.56	(0.48, 0.64)	0.61	(0.53, 0.70)
***≥50***	0.41	(0.34, 0.51)	0.49	(0.42, 0.58)	0.40	(0.35, 0.47)	0.40	(0.35, 0.46)
**Risk Category**								
***MSM***	1.00	–	1.00	–	1.00	–	1.00	–
***IDU***	0.81	(0.65, 1.02)	1.32	(1.11, 1.56)	1.51	(1.30, 1.75)	2.29	(2.00, 2.62)
***Hetero***	1.20	(0.92, 1.56)	1.31	(1.06, 1.60)	1.31	(1.09, 1.58)	1.43	(1.20, 1.70)
***MSM/IDU***	0.33	(0.20, 0.54)	0.66	(0.49, 0.88)	0.77	(0.60, 0.98)	1.19	(0.98, 1.45)
***Other***	3.33	(2.75, 4.02)	4.90	(4.19, 5.72)	4.52	(3.90, 5.23)	3.89	(3.38, 4.47)
**Regional Health Authority**								
***Vancouver Coastal***	1.00	–	1.00	–	1.00	–	1.00	–
***Interior***	0.86	(0.62, 1.20)	1.20	(0.95, 1.53)	1.20	(0.96, 1.50)	1.53	(1.25, 1.88)
***Fraser***	1.06	(0.89, 1.26)	1.18	(1.03, 1.35)	1.133	(1.00, 1.29)	1.12	(1.00, 1.26)
***Vancouver Island***	0.86	(0.66, 1.10)	0.99	(0.82, 1.19)	0.99	(0.83, 1.17)	1.29	(1.10, 1.50)
***Northern***	1.84	(1.33, 2.55)	1.82	(1.40, 2.37)	1.81	(1.40, 2.33)	2.20	(1.70, 2.85)

aOR  =  adjusted odds ratio; CI  =  confidence interval; Risk group categorized as men who have sex with men (MSM), having a history of injection drug use (IDU), both MSM and IDU (MSM/IDU), heterosexual (hetero), or other (blood transfusion or perinatal exposure or unknown risk group (i.e., risk factor not requested or identified)).

Among those retained, the odds of not being initiated on HAART, after controlling for all other factors, were significantly higher for females (compared with males), those <30 (compared with 30–39 year olds), IDUs, heterosexuals, and those in the other risk category (compared with MSM) and residents of the Northern HA (compared with residents of the Vancouver Coastal HA), but significantly lower for MSM/IDUs (compared with MSM) and 40–49 and ≥50 years old (compared with those 30–39 years old). Among those on HAART, the odds of not being suppressed (after controlling for all other factors, were significantly higher for females, individuals <30 (compared with 30–39 year olds), IDUs, heterosexuals and those in the other risk category (compared with MSM), and those residing in the Interior, Vancouver Island and Northern HAs (compared with those residing in Vancouver Coastal HA). The odds of not being suppressed were significantly lower for those 40–49 and ≥50 years old (compared with those 30–39 years old).

## Discussion

In this study, we found that extensive heterogeneity exists in how subgroups and geographic health regions experience attrition along the HIV cascade of care in BC, Canada. We found that 85% of individuals on HAART in BC achieved virologic suppression in 2011; however, this proportion ranged from 60%–93% in our various stratifications. We also found that transitions most vulnerable to leakage were linked to retained and on HAART to suppressed; however, the degree of attrition varied significantly by subgroup and regional health authority. These results are informative and timely, as increasing attention and efforts are focused on the use and implementation of expensive TasP programs in BC and elsewhere [33], [34], [35], [36]. Understanding how to effectively plan and implement such programmatic strategies relies on a strong understanding of the HIV epidemic at a sub-population level.

Since July 2010, HAART has been recommended in B.C. to all HIV-positive individuals, regardless of CD4 cell count with the exception of elite controllers and long-term non-progressors. Thus, it is expected that attrition between the stages retained and on HAART is not a result of treatment guidelines but rather physician and patient-related factors which require further understanding. The largest level of attrition was consistently observed between the stages on HAART to suppressed, likely due to incomplete HAART adherence, suggesting a need to provide greater HAART adherence support.

We observed that females consistently experienced greater cascade attrition than males, with 87% of males on HAART achieving virologic suppression versus only 79% of females on HAART. Similar findings have also been observed in other studies [37], [38], [39]. However, Hall and colleagues, who stratified the cascade using national United States of America HIV surveillance data, found that a greater portion of females were diagnosed and retained in care than males (49% of females vs. 43% of males); however, more males than females were prescribed HAART (90% of males vs. 86% of females) and achieved virologic suppression (80% of males vs. 73% of females) [25]. We hypothesized that the increased attrition among females in our setting may be driven by socio-demographic characteristics (e.g., ethnicity [40], [41], stigma [42], socioeconomic status [43], mental health status [44], competing interests [45]) not fully captured in these analyses. Additionally, females were predominantly IDUs; and when we stratified IDU by gender, we also observed that IDU females experienced greater attrition than IDU males. Our findings suggest a need to review the delivery of female-specific HIV services, as well as further efforts to promote and support adherence and virologic suppression to optimize the benefit of HAART.

It is important to highlight that, regardless of gender or transmission risk category, individuals under 30 years of age experienced the highest rates of attrition. The greatest attrition in this age group occurred between the stages linked to retained (25% loss), retained to on HAART (23% loss) and on HAART to suppressed (27% loss). Similar results have been reported elsewhere with HIV-positive youth having delayed entry and retention in HIV care as well as low adherence to treatment after HAART initiation, resulting in low rates of viral suppression [25], [26], [28], [39], [46], [47], [48]. Factors contributing to poor HIV care engagement and HAART adherence among youth include HIV stigma and disclosure, poor provider-patient relationships, and structural barriers (such as limited access to treatment and inadequate housing) [49], [50]. We observed that the greatest attrition among youth consistently occurred between the stages ‘on HAART’ to ‘suppressed’. The low proportion achieving virologic suppression is likely due to poor HAART adherence. Understanding drivers of poor HAART adherence among youth is necessary to develop HAART adherence improvement interventions. A United States of America focus group investigation of twenty-five 13–24 year olds, found that 50% of participants missed HIV doses out of fear that HIV status would be disclosed to family or friends [50]. While more programmatic research is needed to discover effective youth adherence interventions, two clinical trials have shown that motivational interviewing as well as motivational readiness to initiate HAART improves HAART adherence in this group [50], [51].

The subgroup with the highest rates of attrition was IDUs. Unfortunately, there are several existing barriers that may compromise IDUs uptake of HIV care and their ability to remain engaged in care and sustain viral suppression. These include co-occurring mental health conditions, incarceration episodes, lack of social support, active drug use, homelessness or unstable housing and food insecurity [52], [53], [54], [55], [56]. Research suggests that access to treatment for substance abuse and mental health conditions can improve HAART adherence among HIV-positive IDUs [52], [57], [58]. While BC offers IDU-specific health services such as needle exchange programs, opioid substitution treatment and a supervised injection facility, these services are not available province-wide and they are predominantly located in larger urban areas, particularly within metropolitan Vancouver.

Interestingly, the MSM/IDU group experienced the lowest levels of attrition of all risk groups between the stages diagnosed through on HAART. However, they experienced relatively high attrition from the ‘on HAART’ to ‘suppressed’ cascade stages (16% loss). This finding is in contrast to what was observed by Muthulingam et al. who found that MSM/IDUs had the poorest cascade retention of all risk groups [26]. Our finding may be a result of heightened support and services available to MSM in our setting. The drop in the proportion achieving virologic suppression may be a reflection of challenges specific to IDUs as described above.

We observed that some HAs experienced greater levels of attrition than others. In BC, HIV-care is managed by HAs who have unique patient population characteristics. In some HAs the HIV epidemic is driven by MSM and others by IDU, which is the case of Northern HA. HAs also vary in the number of actively treating physicians and distance to HIV care. In addition, in smaller communities, patient and community-level factors such as fear of HIV identity disclosure to the community, HIV stigma, privacy breaches, may deter a person from being retained in HIV care or initiating HAART [39], [59]. Lastly, HAs that experienced less attrition, like Vancouver Coastal HA, have large urban centres granting access to increased HIV resources (i.e. higher density of physicians and medical care facilities).

There are several limitations to how our cascade was constructed. First, we do not have access to group specific HIV prevalence estimates. Second, as our study population was partially informed using health administrative data, misclassification may have occurred. Third, risk category data was missing for approximately 20% of study participants. Better reporting for the risk category would improve our understanding and interpretation of the cascade by risk group and identify areas for targeted inventions where they need the most help to improve. Fourth, we defined IDUs as those ever having a history of injection drug use, as opposed to current users. Finally, we do not have access to key socio-demographic characteristics, such as income, education or ethnicity, which have been previously shown to have an effect on HAART uptake and outcomes in our setting and elsewhere [60], [61], [62].

In summary, we found that extensive heterogeneity exists in how subgroups and regional health authorities experience attrition along the HIV cascade of care in BC, Canada. Stratifying the cascade allowed us to identify subgroups that are particularly vulnerable to leakage along the cascade. Understanding where attrition is occurring, should allow public health officials to better strategize interventions to meet the needs of specific HIV subgroups.
